# Airborne Bacterial Communities in Three East Asian Cities of China, South Korea, and Japan

**DOI:** 10.1038/s41598-017-05862-4

**Published:** 2017-07-17

**Authors:** Jae Young Lee, Eun Ha Park, Sunghee Lee, GwangPyo Ko, Yasushi Honda, Masahiro Hashizume, Furong Deng, Seung-muk Yi, Ho Kim

**Affiliations:** 1 Institute of Health and Environment and Graduate School of Public Health, Seoul National University, 1, Gwanak-ro, Gwanak-gu, Seoul, 08826 South Korea; 2 KoBiolabs, Inc., 1, Gwanak-ro, Gwanak-gu, Seoul, 08826 South Korea; 3 N-BIO, 1, Gwanak-ro, Gwanak-gu, Seoul, 08826 South Korea; 4 Center for Human and Environmental Microbiome, Institute of Health and Environment, Seoul National University, 1, Gwanak-ro, Gwanak-gu, Seoul, 08826 South Korea; 5 Health and Sport Sciences, The University of Tsukuba, 1-1-1 Tennodai (Comprehensive Res Build D), Tsukuba, 305-8577 Japan; 6 Institute of Tropical Medicine, Nagasaki University, 1-12-4 Sakamoto, Nagasaki, 852-8523 Japan; 7 Department of Occupational & Environmental Health Sciences, Peking University School of Public Health, No. 38 Xueyuan Road, Beijing, 100191 China

## Abstract

The global diversity of airborne bacteria has not yet been studied, despite its importance in human health and climate change. Here, we focused on the diversity of airborne bacteria and their correlations with meteorological/environmental conditions in China, South Korea, and Japan. Beijing (China) had more diverse airborne bacteria, followed by Seoul (South Korea) and Nagasaki (Japan), and seasonal variations were observed. Beijing and Seoul had more diverse airborne bacteria during the winter, whereas Nagasaki showed greater diversity during the summer. According to principal component analysis and Bray-Curtis similarity, higher similarity was observed between Beijing and Seoul than between Seoul and Nagasaki during all seasons except summer. Among meteorological/environmental variables, temperature and humidity were highly correlated with the diversity of airborne bacteria on the measurement day, whereas wind speeds and the frequency of northwest winds were highly correlated for 2–3-day moving averages. Thus, proximity and resuspension could enhance bacterial diversity in East Asian cities.

## Introduction

Studies of airborne microorganisms, abundant components in atmospheric aerosols, have been conducted to elucidate their diversity and possible effects on human health^1–6^. Moreover, because airborne microorganisms can act as cloud condensation nuclei, these organisms may play an important role in global climate change^2, 3, 7–10^. Previous studies have reported the diversity of airborne microorganisms at certain locations. For example, Cao *et al*.^11^ analysed the microbial components of particulate matter in Beijing, China using next-generation sequencing. In their study, the most abundant phyla were *Actinobacteria*, *Proteobacteria*, *Chloroflexi*, *Firmicutes*, *Bacteroidetes*, and *Euryarchaeota*. The most abundant species was *Geodermatophilus obscurus*, a common soil-associated microorganism. Bowers *et al*.^12^ measured airborne bacterial communities at a high-elevation measurement site in northern Colorado in the United States of America (USA) and reported that bacterial abundances depended on the season, with the highest abundances observed in the spring and fall. They also showed that bacterial concentrations increased when total particle concentrations increased. Consistent with Bowers *et al*.^12^, Bertolini *et al*.^13^ reported seasonal variations in airborne bacteria in an urban area in northern Italy. Another study by Bowers *et al*.^14^ reported that airborne bacterial communities in Colorado, USA were different based on sources environments (land-use type) such as agriculture, suburban and forests, not local meteorological conditions. In their study, the dominant bacterial communities at the phylum level were *Proteobacteria*, *Actinobacteria*, and *Firmicutes*, and their compositions varied over the land-use types. Additionally, Lee *et al*.^15^ measured microbial communities in Seoul, South Korea during Asian dust events and found that *Aquabacterium* sp., *Flavobacteriales bacterium* sp., and *Prevotellaceae bacterium* sp. were present. In contrast, *Propionibacterium* sp., *Bacillus* sp., and *Actinetobacter* sp. were detected in non-Asian dust events. Indeed, in several other studies, the concentrations and populations of bacteria have been known to be increased during sandstorm or dust events^16–18^.

Although previous studies have reported the diversity of airborne bacterial communities at certain locations, few longitudinal and multilocation studies have been performed. Only Bowers *et al*.^19^ investigated the diversity of airborne bacterial communities at the continental scale. Bowers *et al*.^19^ collected samples from one small town (Mayville) and three metropolitan cities (Chicago, Detroit, and Cleveland) in the Midwestern USA and found that the diversity of bacterial communities depended on the location and season.

Despite the importance of airborne bacterial communities in the atmospheric environment, no studies have reported the diversity of airborne bacteria in multiple countries and/or on the global scale. Such studies may help to elucidate variations between adjacent locations and the movements of microorganisms over the land and sea. Thus, in this study, we measured airborne bacteria in three major cities in three countries of East Asia (Beijing in China, Seoul in South Korea, and Nagasaki in Japan). This study is important for three major reasons. First, we used a new sequencing method (amplicon-based sequencing) using Illumina Miseq, which detected both cultivatable and noncultivatable microorganism populations^11^. Since the recent development of the new sequencing method a decade ago, it has been widely adopted for studying microbial communities. Second, to the best of our knowledge, this study is the first to show the airborne bacterial diversity in three different Asian countries using the new sequencing method, for a better understanding of seasonal and regional variations in the bacterial communities. Finally, we also evaluate the correlations between the diversity of airborne bacterial communities and meteorological/environmental factors, such as temperature, humidity, wind speed, wind direction, and particulate matter (PM_2.5_) concentrations.

## Method

### PM sampling and handling

PM_2.5_ concentrations were measured in China, South Korea, and Japan, and measurement systems were installed on the rooftops of the School of Public Health buildings at Peking University in Beijing, Seoul National University in Seoul, and Nagasaki University in Nagasaki. The sampling locations were chosen since the researchers from the three universities were in collaboration for this study under GRL (Global Research Lab) project, and these universities were located roughly at the middle of each city. Supplementary Figure S1 shows the locations of the measurement sites on a map of East Asia. General information of each measurement site is summarised in Supplementary Table S1. Sampling at each site was conducted using the same type of cyclone (URG-2000-30EH, URG, USA) and filter pack system (URG-2000-30FG; URG). The average flow rates of measurement sites in Beijing, Seoul, and Nagasaki were 16.7 L/min for 24 h on the sampling day. Using these systems, PM_2.5_ was collected onto 47-mm Teflon filters (PTFE membrane filters; PALL Corp., USA). Filters were then packed in aluminium foil and stored at −20 °C until analysis. Collected filter samples were dried and then analysed for gravimetric concentrations using the balance (AND HM-202; AND, Japan) with a resolution of 0.01 mg.

### Collection of meteorological and environmental variables

Meteorological data (e.g. daily mean temperature, relative humidity, wind speed, and wind direction) of Beijing, Seoul, and Nagasaki during the measurement period were obtained from Chinese weather and air pollution query websites (http://www.tianqihoubao.com, http://weatherarchive.ru), Korea Meteorological Administration, and Japan Meteorological Agency, respectively.

### DNA extraction from PM samples

Each PM sample was collected on Whatman paper (Whatman International Ltd., UK) and scraped together using a sterilised toothpick. Total DNA from scraped samples was extracted using a PowerSoil DNA Isolation Kit (MoBio Laboratories, Carlsbad, CA, USA) following the manufacturer’s protocol. The final volume of isolated total DNA was 50 μL in TE buffer (pH 8.0), and samples were stored in a freezer until analysis.

### 16S rRNA gene amplification followed by Illumina Miseq

For each sample, 16S rRNA genes were amplified with Illumina-adapted universal primers (515 F/806 R) for amplification of the V4 region (515 F: forward primer, 5′-AATGATACGGCGACCACCGAGATCTACACTATGGTAATTGTGTGCCAGCMGCCGCGGTAA-3′; 806 R: reverse primer containing a unique 12-base golay barcode for tagging each polymerase chain reaction [PCR] product, 5′-CAAGCAGAAGACGGCATACGAGATNNNNNNNNNNNNAGTCAGTCAGCCGGACTACHVGGGTWTCTAAT-3′). PCR mixtures (50 μL) contained 35.5 μL PCR water, 5 μL 10 × Takara Ex Taq buffer, 0.1 mM Takara dNTP mix, 0.25 μM of each primer, 0.05 U Ex Taq polymerase (TaKaRa, Shiga, Japan), and 5.0 μL genomic DNA. Reactions were held at 94 °C for 3 min for denaturation, followed 35 cycles at 94 °C for 45 s, 50 °C for 60 s, and 72 °C for 90 s, and then a final extension at 72 °C for 10 min to ensure complete amplification. Amplified PCR products were purified using an UltraClean PCR Clean-Up Kit (MO BIO Laboratory, Inc., USA) and quantified using a KAPA Library Quantification Kit (KAPA Biosystems, Woburn, MA, USA). The amplicons for each sample were normalised and pooled. Bacterial 16S rRNA genes were sequenced using a MiSeq Reagent Kit V3 (2 × 300 cycles) with the MiSeq platform (Illumina, San Diego, CA, USA).

### Bioinformatics analysis of 16S rRNA genes

After preprocessing of quality filter (Q > 20) and trimming (removing adaptor and primers) steps using a FastX-toolkit, the sequences were assigned to operational taxonomic units (OTUs; 97% identity) using the Greengenes database (gg_13_5), followed by selection of representative sequences using Quantitative Insights Into Microbial Ecology (QIIME 1.8.0)^20^. A chimeric check was performed and 15% of reads were dropped during this process. The final 16S rRNA genes of the air samples yielded 906,573 reads.

Microbial classification based on 16S rRNA gene sequences was performed using the ribosomal database project (RDP) classifier naïve Bayesian algorithm^21^. Taxonomic identities of the phylotypes were assigned using RDP taxonomic annotations. Complete sequences were aligned by nearest-alignment space termination (NAST) with greater than 75% identity based on a nonchimeric core set of at least 1,250 nt in length^22^ and filtered by lanemask to remove columns comprising only gaps^23^ before building the tree. Phylogenetic trees were produced using the FastTree method.

### Data availability

The sequences from this study were deposited in the European Nucleotide Archive under the study accession number PBJEB18728. The data sets that support the findings of this study are available from the first author on reasonable request.

## Results

### Relative abundance of airborne bacteria

**Figure 1:**
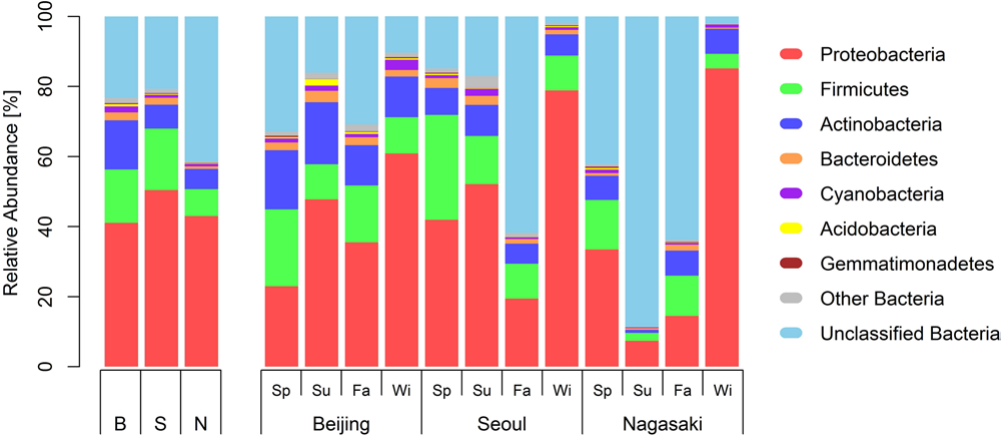
Relative abundance of airborne bacteria at the phylum level at each measurement site during the measurement period. B, S, and N indicate the Beijing, Seoul, and Nagasaki sites, respectively, and Sp., Su., Fa., and Wi. indicate the four seasons (spring, summer, fall, and winter, respectively).

In this study, we analysed the relative abundance and diversity of microbial communities in three cities in China, South Korea, and Japan during the measurement period. Figure 1 shows the relative abundances of airborne bacteria at the phylum level at each measurement site in each season, and Supplementary Table S2 summarises the relative abundances averaged for all sites and all seasons. From these results, *Proteobacteria* were the most abundant bacteria at the phylum level, comprising approximately 44.5% of the total airborne microorganisms, followed by *Firmicutes* (13.6%) and *Actinobacteria* (9.2%). Supplementary Table S3 compares the relative abundances in the three sites. The top six abundant bacteria in the three sites were identical, and the compositions of the airborne microorganism communities in the three sites were similar. However, the Beijing site had a relatively high level of *Actinobacteria* compared with the other two sites, and the Seoul site had a relatively high level of *Firmicutes*. Additionally, the Nagasaki site had a relatively high level of unclassified bacteria.

As shown in Fig. 1 and Supplementary Table S4, the composition of airborne microorganisms varied greatly at the phylum level from season to season. This seasonal variation in the microorganism composition was greater than the spatial variation among the three sites. This trend was particularly prominent in the Seoul and Nagasaki sites, whereas the Beijing site showed relatively less variation over the seasons. For example, *Proteobacteria* in Seoul comprised 19.4% of total microorganisms in the fall but 78.8% in the winter. Similarly, the portion of *Proteobacteria* decreased to 7.4% in the summer and increased to 85.1% in the winter in Nagasaki.

**Figure 2:**
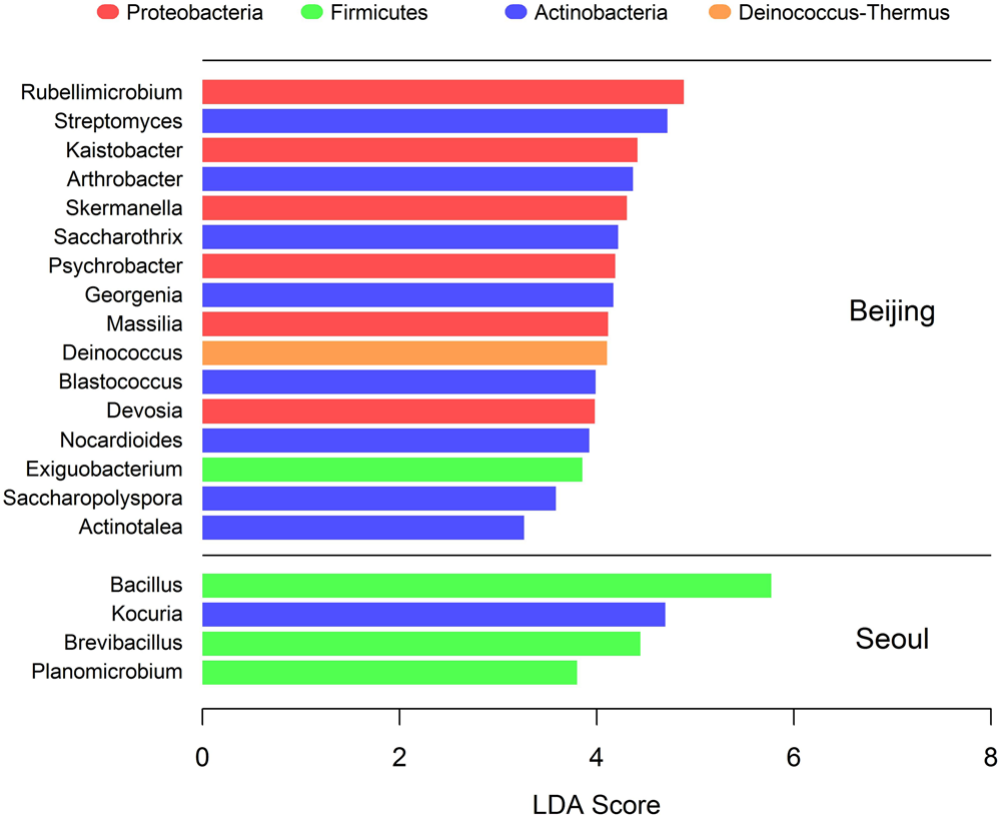
The results of LEfSe analysis, which identified bacterial genera that were significantly abundant in one site compared with the other two sites. Bacterial genera with the LDA score of more than 3 are shown. Colours indicate the phylum level categories of these bacteria.

To identify specific bacterial genera that were significantly abundant in one site than the others, LEfSe (LDA Effective Size) analysis^24^ was performed. Figure 2 shows the results of LEfSe analysis. Based on the LDA (Linear Discriminant Analysis) score, 16 genus-level bacteria were found to be significantly more abundant in the Beijing site than in the Seoul and Nagasaki sites. The top three bacterial genera based on LDA scores were *Rubellimicrobium*, *Streptomyces*, and *Kaistobacter*. Similarly, four bacteria were found to be much more abundant in the Seoul site than in the other two sites. The top three genera based on LDA scores were *Bacillus*, *Kocuria*, and *Brevibacillus*. No significantly abundant genus-level bacteria were found in the Nagasaki site. Categorisation of these 20 bacteria based on phyla showed that among the 16 bacteria abundant in the Beijing site, six were *Proteobacteria*, eight were *Actinobacteria*, one was *Deinococcus*-*Thermus*, and one was *Firmicutes*; among the four bacteria abundant in the Seoul site, three were *Firmicutes*, and one was *Actinobacteria*. Note that the largest number of abundant genera were categorized as *Actinobacteria* in the Beijing site (eight out of sixteen) and as *Firmicutes* in the Seoul site (three out of four). This result coincides with the phylum-level abundance analysis in that the Beijing and Seoul sites showed relatively high levels of *Actinobacteria* and *Firmicutes*, respectively (see Fig. 1 and Supplementary Table S3).

### Diversity of airborne bacteria

**Figure 3:**
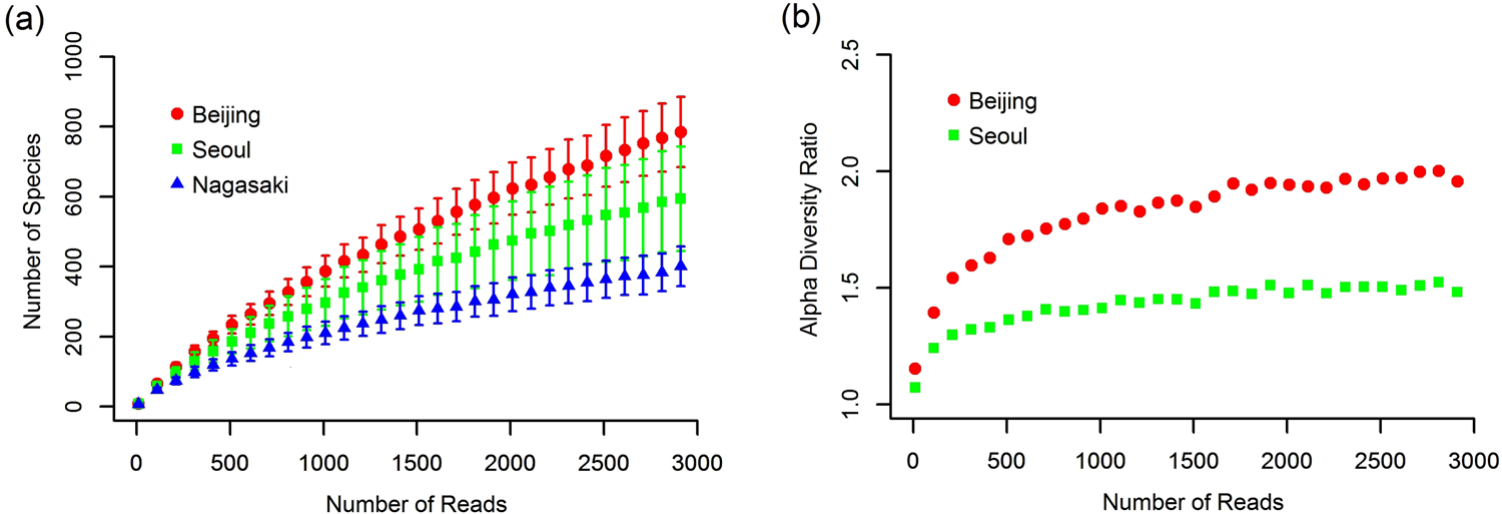
(**a**) Diversity versus number of reads at the species level at each measurement site. Red circles, green rectangles, and blue triangles represent the numbers of species in the Beijing, Seoul, and Nagasaki sites, respectively. (**b**) Diversity of the Beijing (red circles) and Seoul (green rectangles) sites divided by that of the Nagasaki site.

Figure 3(a) shows alpha diversity accumulation curves showing how the number of locally found species increased as the number of reads increased in each measurement site. The average number of observed species in Beijing samples increased fastest among the three cities as the number of reads increased, followed by those in Seoul and Nagasaki samples. Thus, the Beijing site had the most diverse local species, followed by the Seoul and Nagasaki sites. Figure 3(b) shows the alpha diversity ratios of Beijing and Seoul compared with that of Nagasaki. These ratios were obtained by dividing the species richness of the two sites by that of Nagasaki. From this analysis, we found that the Beijing and Seoul sites had about 2 and 1.5 times more alpha diversity than the Nagasaki site after the diversity ratio became saturated.

**Figure 4:**
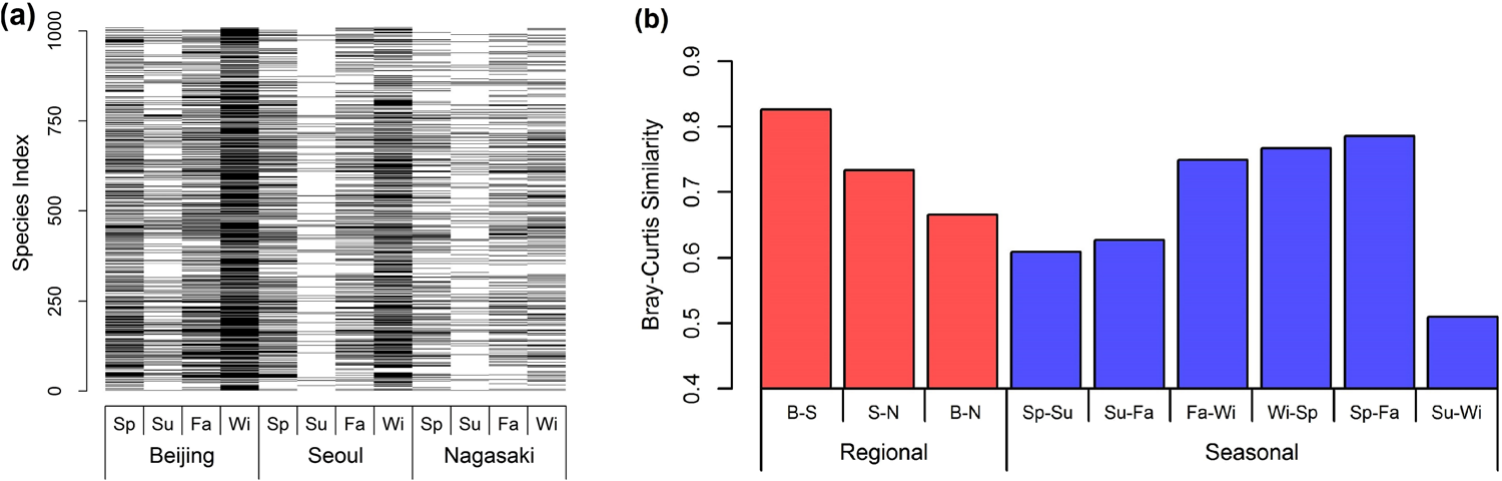
(**a**) Heat map of the identified phylotypes at the genus level, where identified phylotypes are in black and unidentified phylotypes are in white. Sp., Su., Fa., and Wi. indicate the four seasons (spring, summer, fall, and winter), respectively. (**b**) Bray-Curtis similarities of the identified phylotypes at the genus level between measurements sites and between seasons. B-S, S-N, and B-N represent comparisons between Beijing and Seoul, Seoul and Nagasaki, and Beijing and Nagasaki, respectively. Sp., Su., Fa., and Wi. indicate the four seasons, respectively.

Figure 4(a) shows a heat map of the identified species at the genus level in each location and provides an alternate way for visualising the diversity of the identified species. As can be seen, the Beijing heat map shows the largest number of black lines, each of which corresponds to an identified phylotype, followed by those of Seoul and Nagasaki. This indicates the highest diversity in Beijing samples. In addition, Fig. 4(a) shows how the heat map varies from season to season. Note that all three cities showed similar seasonal variation patterns, i.e., highest diversity during winter and lowest diversity during summer. To quantitatively understand the regional and seasonal variations in bacterial diversity, the similarities between the heat maps were measured based on the Bray-Curtis similarity index. Figure 4(b) visualizes the Bray-Curtis similarities of the bacterial communities. The regional Bray-Curtis similarity between Beijing and Seoul was 0.83, indicating that 83% of the average number of total species found in Beijing and Seoul were common in both cities. The Bray-Curtis similarity between Seoul and Nagasaki was 0.73 and that between Beijing and Nagasaki was 0.66. The bacterial communities of Beijing and Seoul were much more similar to each other than any other two cities. Figure 4(b) also shows the seasonal similarity. The highest similarity was observed between spring and fall (0.79), while the lowest similarity was observed between summer and winter (0.51). Judging from the similarity index, the seasonal variations in bacterial communities were higher than the regional variations even though the measurement locations were in three different countries. Supplementary Figure S2 shows the seasonal variations in similarity. Similarity between Beijing and Seoul was high in the spring and winter and low in the summer, whereas that between Seoul and Nagasaki was high in the spring and fall and low in the summer. Interestingly, the similarity between Beijing and Seoul was higher than that between Seoul and Nagasaki during all seasons except summer.

**Figure 5:**
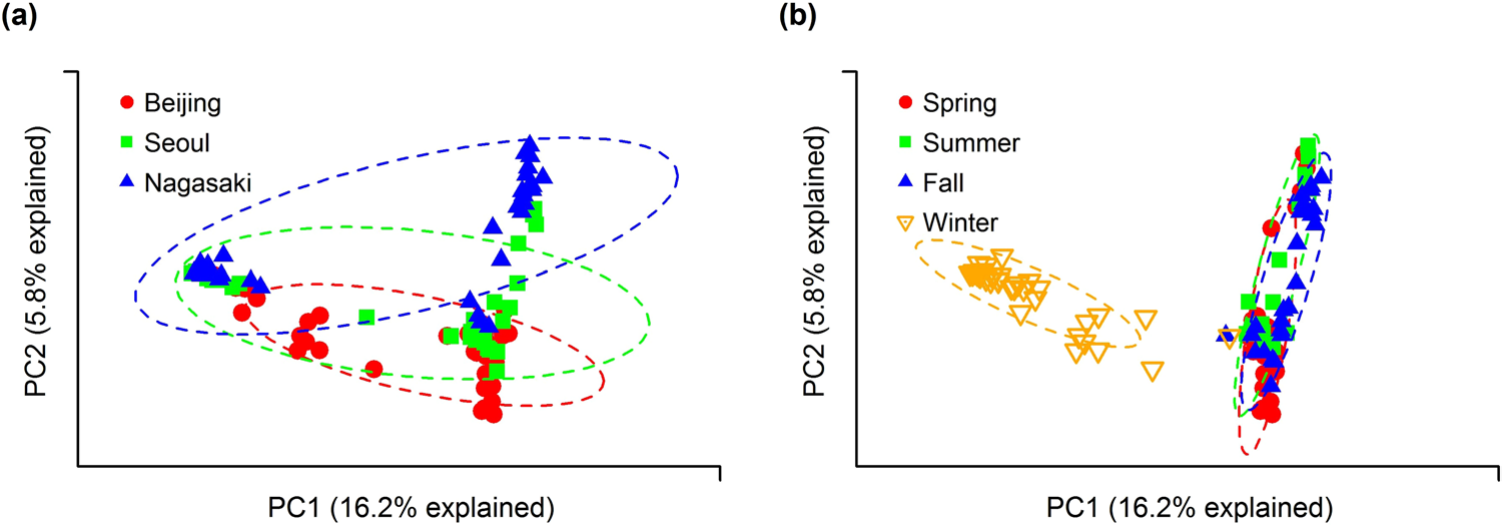
Principal component analysis (PCA) at the OTU level based on the Bray-Curtis distance for all measurement samples. Samples are grouped (**a**) by city and (**b**) by season.

To understand the compositional differences or similarities, principal component analysis (PCA) was conducted for all measured samples. In Fig. 5, Beijing, Seoul, and Nagasaki samples are depicted in the two-dimensional space defined by the first and second principal components, and ellipses were drawn to indicate the position at which each city’s samples were clustered. The analysis of similarities (ANOSIM) was performed and gave the R value of 0.173 and the p-value of 0.001. Notably, Beijing and Nagasaki samples were clustered farther from each other, and Seoul samples were clustered in between, indicating that the bacterial communities of Beijing and Nagasaki were more different than those of Beijing and Seoul or Seoul and Nagasaki. These results can be partly explained by the geographical locations of cities (see Supplementary Table S1 for the coordinates of three locations). Seoul is geographically located between Beijing and Nagasaki. It is closer to Beijing in terms of latitude, while closer to Nagasaki in terms of longitude. When the samples were grouped by season (see Fig. 5(b)), spring, summer, fall samples were clustered together, while winter samples were separated from other samples (ANOSIM R = 0.601, p-value = 0.001). This indicates that the winter samples harbored bacterial communities distinct from others.

### Correlations between diversity and environmental factors

**Figure 6:**
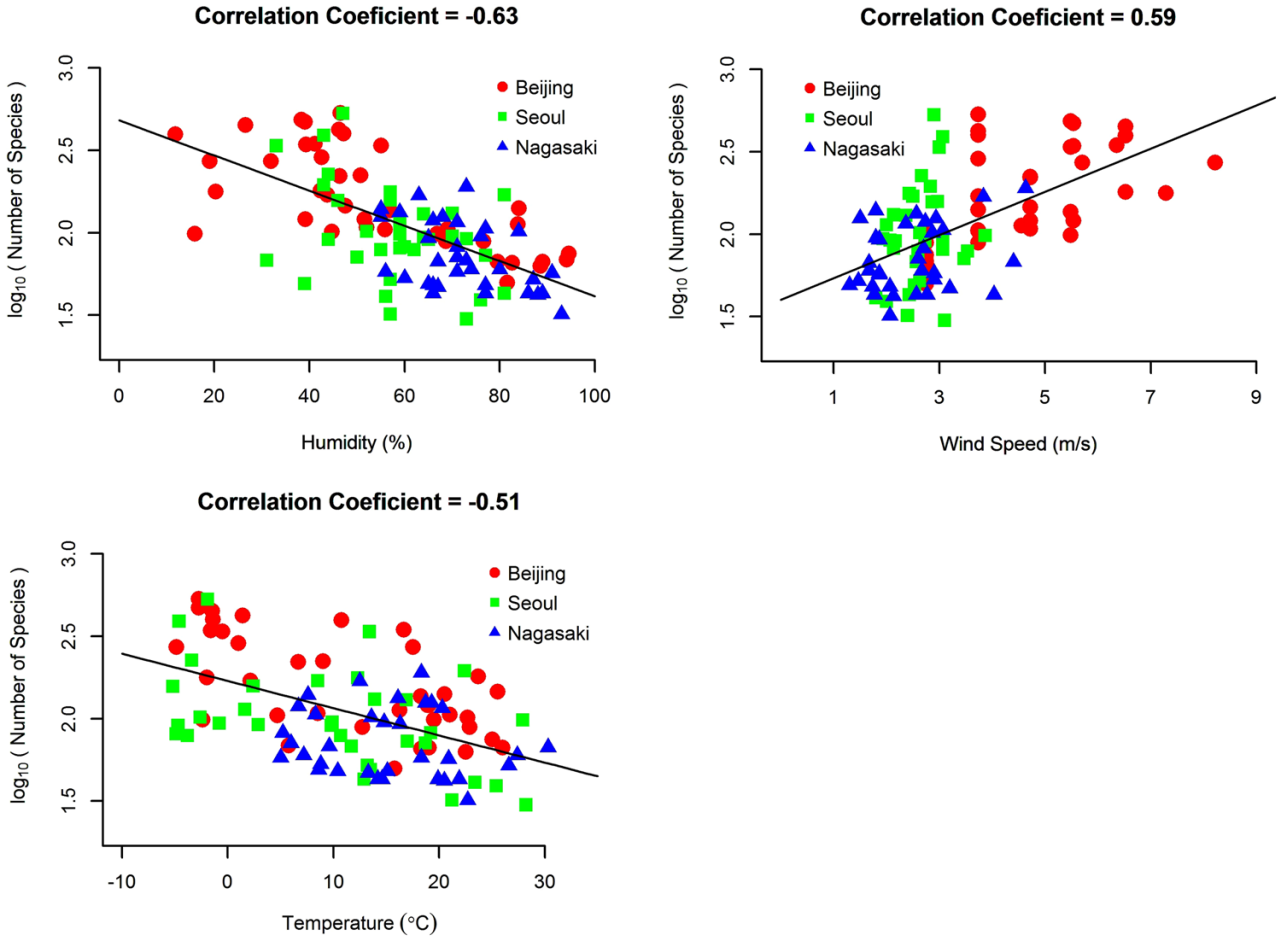
Scatter plots showing the correlation between airborne bacterial diversity and meteorological factors, such as humidity, wind speed, and temperature.

According to the data shown above, the diversity of airborne bacteria had strong spatial and seasonal variations. In order to identify the factors that affect the diversity of airborne bacteria, we first gathered environmental and meteorological information. Supplementary Table S5 summarized the description of collected samples and their corresponding meteorological and environmental conditions on the measurement day. Based on this information, we then calculated the 2-, 3-, and 8-day moving averages for each factor. Then, we calculated the correlation coefficients between the species richness and the daily and moving averaged factors. Table 1 summarises the factors with high correlations, and Fig. 6 shows scatter plots of the data.

**Table 1:** Correlation coefficients between diversity of airborne bacterial communities and environmental/meteorological factors.

	Daily	MA2	MA3	MA8
Humidity (%)	**−0.63**	−0.61	−0.59	−0.50
Wind Speed (m/s)	0.36	0.50	**0.59**	0.56
Temperature (°C)	**−0.51**	−0.49	−0.49	−0.48
Frequency of northwest wind (%)	0.25	**0.34**	**0.34**	0.33
PM_2.5_ (μg/m^3^)^*^	0.09	—	—	—

Note: MA2, MA3, and MA8 represent the 2-, 3-, and 8-day moving averages, respectively.

^*^PM_2.5_ concentrations were measured on a sampling day, thus no moving average was applied.

As shown in Table 1, there was a negative correlation between humidity and the diversity of airborne bacteria. Temperature was also negatively correlated with bacterial diversity, whereas other factors, such as wind speed and frequency of northwest wind were positively correlated. In addition, bacterial diversity was not correlated with the concentration of PM_2.5_. For wind speed and the frequency of northwest wind, the moving average values showed a higher correlation than the daily value, indicating that the diversity was affected by the cumulative effects over a few days. In contrast, for humidity, daily values showed higher correlations, indicating that the condition on the day of measurement was much more important that the cumulative condition during the most recent few days. For temperature, daily values and the moving average values were similar. In addition to the richness index, the Shannon index was also used for the diversity metric to consider both richness and evenness in diversity. The results were shown in Supplementary Table S6 and Supplementary Figure S3. Note that the correlation coefficients with the Shannon index decreased compared to those with the richness index. This can be interpreted that the meteorological conditions that increase the diversity (for example, low humidity, high wind speed, and etc.) mostly introduce unabundant and minor bacteria.

## Discussion

### Regional and seasonal variations in bacterial communities

In this work, we analysed airborne bacterial communities in three measurement locations which were in three East Asian cities (Beijing, Seoul, and Nagasaki). The bacterial communities were compared in terms of relative abundance, diversity, and Bray-Curtis similarity. All of these results consistently demonstrated that there were both regional and seasonal variations and that the seasonal variation was relatively larger than the regional variation (see Figs 1 and 4(b) for the result). This result is interesting because the three locations are in different country, they are very far from each other (952 km from Beijing to Seoul, 596 km from Seoul to Nagasaki, and 1442 km from Beijing to Nagasaki), and the environments near them are different, but yet the seasonal variation is greater. This is consistent with a previous study by Bowers *et al*.^19^, who showed that the compositions and concentrations of airborne bacteria in Midwestern cities in the USA varied by both season and region, albeit with greater seasonal than regional variations. Bowers *et al*.^12^ and Bertolini *et al*.^13^ also separately reported seasonal variations in airborne bacteria in their papers.

However, there was an important difference between the previous findings and our current results. Specifically, we found that the diversity of airborne bacteria increased in the winter and decreased in the summer, in contrast to a previous study^19^ in which the authors showed that the concentration of airborne bacteria decreased in the winter and increased in the summer in Midwestern cities in the USA. They explained that there is a limited number of sources of airborne bacteria in winter because plants and trees typically becomes leafless, the ground may be covered with snow, and water may be frozen, thereby decreasing the concentration of airborne bacteria. In another study conducted by the same authors, they reported that bacterial abundances depended on the season, with the highest diversity observed in the fall and the lowest diversity observed in the winter in northern Colorado in the USA^12^. Nonetheless, East Asian cities exhibited high bacterial diversity in the winter. This could be explained by the unique meteorological characteristics of these regions, e.g., the East Asian monsoon, in which cold and dry wind flows from the Siberian region to the Pacific Ocean (northwest) in the winter, whereas hot and humid wind flows from the ocean to Siberia (southeast) in the summer^25^. As a result, the three cities experience the air from the continent in the winter and from the ocean in the summer. The continental air mass is expected to be influenced by more diverse sources of bacteria than the oceanic air mass. Moreover, the greater bacterial diversity in the winter can also be explained by the consistency of the wind direction throughout the season.

In addition, meteorological conditions in winter showed low humidity and high winds, which may contribute to the suspension of ground bacteria into airborne bacteria. Since suspension of ground bacteria is one of the major sources of airborne bacteria, environmental conditions that facilitate this suspension also increase the diversity of airborne bacteria. Less humidity helps the suspension because dry particles are lighter than wet particles, and faster wind also helps the suspension because it has more energy to lift particles into the air. The effects of monsoons and the relationship between climate and suspension of ground bacteria can explain the high correlation between the diversity and meteorological parameters, such as humidity, temperature, and wind speed. The negative correlation with relative humidity is consistent with the previous study conducted by Tong and Lighthart^26^, and the positive correlation with wind speed is consistent with the study by Jones and Harrison^27^. In contrast, Bowers *et al*.^12^ argued no strong correlations were found between the airborne bacterial concentrations and the meteorological factors.

### Limitations

In this study, we directly measured PM_2.5_ concentrations and analysed the airborne bacterial communities by ourselves, while the environmental and meteorological information was obtained from government organizations or public data sources as described in the method section. The possible problem of using the public meteorological data stems from the fact that the measurement locations of meteorological parameters are different from those of airborne bacteria, and the measurement process may not be the same for all locations. This may underestimate the correlation between the bacterial diversity and meteorological parameters. Second, the number of samples were not consistent in each location and each season (see Supplementary Table S5). Therefore, the sampling variations may not be consistent for all locations and seasons in the analysis. Lastly, the measurements were taken place at the rooftops of the School of Public Health buildings in three universities. This choice leaded to the measurement heights not being the ground level nor identical for three locations (see Supplementary Table S1). It should be noted that the regional variations analysed in this study included the portion introduced by the height difference if any. Also, care should be taken when referring the finding in this research for the human health in the ground level. Further study is needed to identify the vertical variations of bacterial community.

### Future work and challenges

Airborne bacteria are known to have adverse effects on human health. However, little research on airborne bacteria has been published, and more in-depth analyses are needed. First, the pathogenicity of abundant bacteria must be studied. This can be performed in a hospital environment, either through cohort studies or experiments in animals. Time-series analyses may also contribute to our understanding of the pathogenicity of airborne bacteria. However, this method typically requires daily sequencing of atmospheric samples, which is often limited by sampling and sequencing capacity for species-level characterisation. Second, the source of airborne bacteria must be understood. This is necessary for eventually controlling and reducing the concentrations of harmful bacteria through policy making and urban planning. Elucidation of the sources of airborne bacteria can be carried out by backward trajectory and source apportionment analyses. However, these methods also require sequencing of large numbers of samples. Moreover, bacteria may reproduce while traveling, making them more difficult to analyse. Third, multiple locations must be sampled and analysed at each city for a systematic surveying of the city’s airborne microbial community.

## Conclusions

In this study, we analysed airborne bacteria at the species level from 108 samples collected in three Asian cities (Beijing, Seoul, and Nagasaki). Relative abundance and LEfSe analyses identified significantly abundant bacteria in each city. Diversity analysis revealed that Beijing had the most diverse bacterial community among the three cities, followed by Seoul and Nagasaki. PCA showed that the bacteria community in Seoul was in between those of Beijing and Nagasaki, and Bray-Curtis similarity analysis demonstrated that airborne bacteria in Seoul were more similar to those in Beijing than those in Nagasaki, except during summer. Taken together, these analyses consistently showed that there were regional and seasonal variations in bacterial communities and that the seasonal variation was relatively larger. Finally, we also showed that there were correlations between the diversity of airborne bacteria and meteorological variables, such as relative humidity, temperature, and wind speed. The results and findings of this study provide a useful reference for future health studies, policy making, and urban planning.

## Electronic supplementary material

Supplementary material
