# Supplementary material for: Airborne Bacterial Communities in Three East Asian Cities of China, South Korea, and Japan
Source: Sci Rep. 2017 Jul 17;7:5545. doi: 10.1038/s41598-017-05862-4 (PMC5514139; doi:10.1038/s41598-017-05862-4)
Supplement: Supplementary file 1 — Supplementary material [file 41598_2017_5862_MOESM1_ESM.doc]

**Airborne Bacterial Communities in Three East Asian Cities of China, South Korea, and Japan**

Jae Young Lee^1^, Eun Ha Park^1^, Sunghee Lee^2,3^, GwangPyo Ko^1,2,3,4^, Yasushi Honda^5^, Masahiro Hashizume^6^, Furong Deng^7^, Seung-muk Yi^1^, Ho Kim^1,*^

^1^Institute of Health and Environment and Graduate School of Public Health, Seoul National University

1, Gwanak-ro, Gwanak-gu, Seoul 08826, South Korea

E-mail (J.Y. Lee): jaeyoung.lee@alumni.stanford.edu

Tel (J.Y. Lee): +82-10-2759-9135

Fax: +82-2-745-9204

^2^KoBiolabs, Inc., 1, Gwanak-ro, Gwanak-gu, Seoul 08826, South Korea

Tel: +82-2-888-9939

Fax: +82-2-888-9940

^3^N-BIO, 1, Gwanak-ro, Gwanak-gu, Seoul 08826, South Korea

^4^Center for Human and Environmental Microbiome, Institute of Health and Environment, Seoul National University, 1, Gwanak-ro, Gwanak-gu, Seoul 08826, South Korea

^5^Health and Sport Sciences, The University of Tsukuba

1-1-1 Tennodai (Comprehensive Res Build D), Tsukuba 305-8577 Japan

Tel: +81 29 853 2627

Fax: +81 29 853 3255

^6^Institute of Tropical Medicine, Nagasaki University

1-12-4 Sakamoto, Nagasaki 852-8523, Japan

Phone: +81 (0)95 819 7763

Fax: +81 (0)95 819 7844

^7^Department of Occupational & Environmental Health Sciences, Peking University School of Public Health

No. 38 Xueyuan Road, Beijing 100191, China

Tel: +86-13141335378

E-mail: lotus321321@126.com

^*^*Corresponding author:* Ho Kim, Professor, Dean

Graduate School of Public Health and Asian Institute for Energy, Environment & Sustainability, Seoul National University

1, Gwanak-ro, Gwanak-gu, Seoul 08826, South Korea

E-mail: hokim@snu.ac.kr

Tel: +82-2-880-2711

Fax: +82-2-745-9204


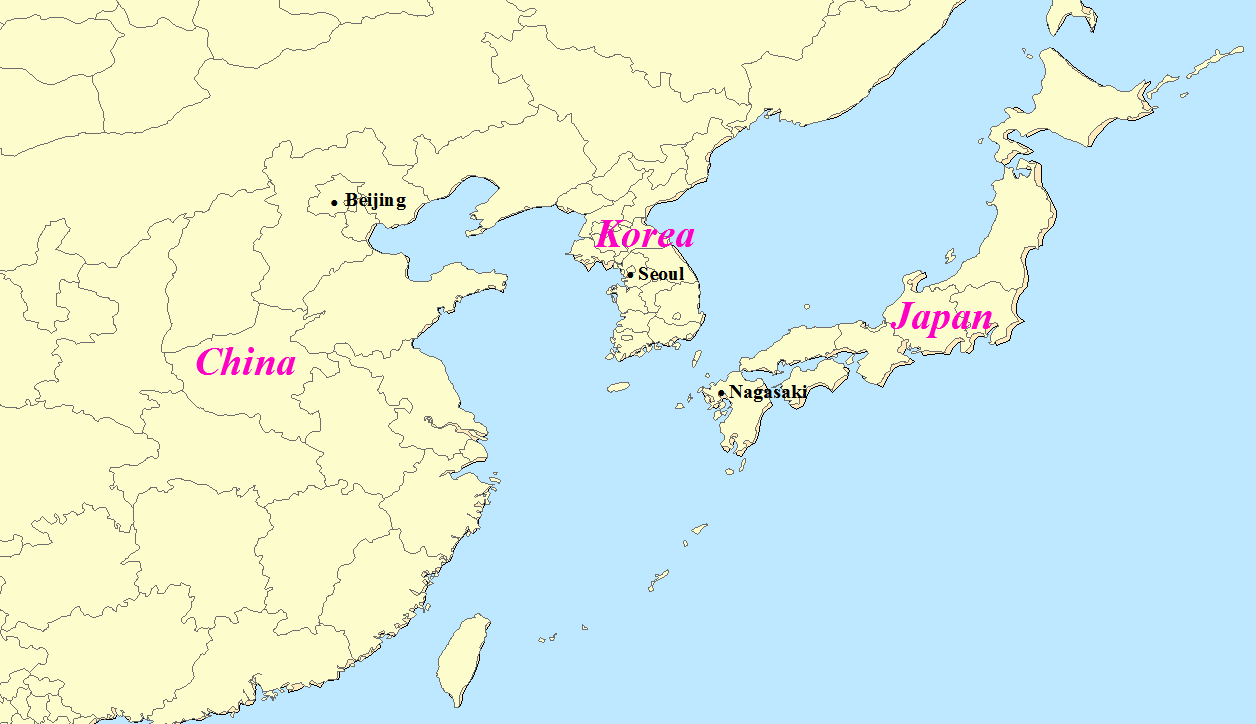


Figure S1. A map of East Asia and the locations of three measurement sites. This map was generated by using ArcGIS 10 Desktop (http://desktop.arcgis.com/en/).

Table S1. General measurement information of each measurement site

|  | | **Beijing site** | **Seoul site** | **Nagasaki site** |
| --- | --- | --- | --- | --- |
| **Measurement date** | | 12/02/2014 ~ 11/11/2015 | 12/04/2014 ~ 11/23/2015 | 12/01/2014 ~ 11/05/2015 |
| **Number of samples** | | 39 | 33 | 33 |
| **Average read depth / sample** | | 10314 | 7555 | 7720 |
| **Measurement**  **location** | **Country** | China | South Korea | Japan |
|  | **City** | Beijing | Seoul | Nagasaki |
|  | **University** | Peking University | Seoul National University | Nagasaki University |
|  | **Coordinates** | 39.96 N, 116.30 E | 37.514 N, 127.0 E | 32.77 N, 129.87 E |
|  | **Height*** | 7^th^ floor (~21m) | 5^th^ floor (~15m) | 4^rd^ floor (~12m) |

* Measurement heights in meter from the ground level were calculated based on multiplying the floor level by 3.

Table S2. Relative abundance of airborne microorganism in phylum level for all cities and all seasons

|  | All Cities, All Seasons | |
| --- | --- | --- |
| Category | Name | Ratio |
| Top 7 Abundant Bacteria | Proteobacteria | 44.5% |
|  | Firmicutes | 13.6% |
|  | Actinobacteria | 9.2% |
|  | Bacteroidetes | 1.7% |
|  | Cyanobacteria | 1.1% |
|  | Acidobacteria | 0.4% |
|  | Gemmatimonadetes | 0.2% |
| Other Bacteria | - | 1.1% |
| Unclassified Bacteria | - | 28.2% |

Table S3. Relative abundance of airborne microorganism in phylum level at each city.

|  | Beijing, All Seasons | | Seoul, All Seasons | | Nagasaki, All Seasons | |
| --- | --- | --- | --- | --- | --- | --- |
| Category | Name | Ratio | Name | Ratio | Name | Ratio |
| Top 7 Abundant Bacteria | Proteobacteria | 41.0% | Proteobacteria | 50.4% | Proteobacteria | 43.0% |
|  | Firmicutes | 15.3% | Firmicutes | 17.5% | Firmicutes | 7.7% |
|  | Actinobacteria | 14.0% | Actinobacteria | 6.8% | Actinobacteria | 5.8% |
|  | Bacteroidetes | 2.3% | Bacteroidetes | 2.0% | Bacteroidetes | 0.7% |
|  | Cyanobacteria | 1.7% | Cyanobacteria | 0.8% | Cyanobacteria | 0.7% |
|  | Acidobacteria | 0.7% | Acidobacteria | 0.3% | Acidobacteria | 0.2% |
|  | Chloroflexi | 0.3% | Armatimonadetes | 0.3% | Armatimonadetes | 0.1% |
| Other Bacteria | - | 1.5% | - | 1.1% | - | 0.4% |
| Unclassified Bacteria | - | 23.3% | - | 20.7% | - | 41.4% |

Table S4. Relative abundance of airborne microorganism in phylum level at each city and each season.

|  | Beijing, Spring | | Beijing, Summer | | Beijing, Fall | | Beijing, Winter | |
| --- | --- | --- | --- | --- | --- | --- | --- | --- |
| Category | Name | Ratio | Name | Ratio | Name | Ratio | Name | Ratio |
| Top 7 Abundant Bacteria | Proteobacteria | 22.9% | Proteobacteria | 47.8% | Proteobacteria | 35.5% | Proteobacteria | 60.9% |
|  | Firmicutes | 22.0% | Actinobacteria | 17.7% | Firmicutes | 16.2% | Actinobacteria | 11.6% |
|  | Actinobacteria | 16.9% | Firmicutes | 10.0% | Actinobacteria | 11.5% | Firmicutes | 10.3% |
|  | Bacteroidetes | 2.2% | Bacteroidetes | 3.3% | Bacteroidetes | 2.3% | Cyanobacteria | 3.0% |
|  | Cyanobacteria | 1.2% | Acidobacteria | 1.9% | Cyanobacteria | 0.9% | Bacteroidetes | 1.9% |
|  | Chloroflexi | 0.4% | Cyanobacteria | 1.5% | Acidobacteria | 0.6% | Chloroflexi | 0.4% |
|  | Acidobacteria | 0.4% | Thermi | 0.4% | Thermi | 0.5% | Acidobacteria | 0.4% |
| Other Bacteria | - | 1.3% | - | 1.5% | - | 1.6% | - | 1.2% |
| Unclassified Bacteria | - | 32.8% | - | 16.1% | - | 30.9% | - | 10.4% |

|  | Seoul, Spring | | Seoul, Summer | | Seoul, Fall | | Seoul, Winter | |
| --- | --- | --- | --- | --- | --- | --- | --- | --- |
| Category | Name | Ratio | Name | Ratio | Name | Ratio | Name | Ratio |
| Top 7 Abundant Bacteria | Proteobacteria | 42.0% | Proteobacteria | 52.1% | Proteobacteria | 19.4% | Proteobacteria | 78.9% |
|  | Firmicutes | 29.9% | Firmicutes | 13.8% | Firmicutes | 10.0% | Firmicutes | 9.9% |
|  | Actinobacteria | 7.7% | Actinobacteria | 8.8% | Actinobacteria | 5.7% | Actinobacteria | 6.0% |
|  | Bacteroidetes | 2.8% | Armatimonadetes | 2.8% | Bacteroidetes | 1.3% | Bacteroidetes | 1.3% |
|  | Cyanobacteria | 0.9% | Bacteroidetes | 2.6% | Tenericutes | 0.8% | Cyanobacteria | 0.8% |
|  | Acidobacteria | 0.4% | Cyanobacteria | 2.0% | Cyanobacteria | 0.4% | Acidobacteria | 0.4% |
|  | Fusobacteria | 0.4% | Acidobacteria | 0.2% | Armatimonadetes | 0.1% | Chloroflexi | 0.3% |
| Other Bacteria | - | 1.0% | - | 0.5% | - | 0.5% | - | 0.7% |
| Unclassified Bacteria | - | 14.9% | - | 17.1% | - | 61.8% | - | 1.9% |

|  | Nagasaki, Spring | | Nagasaki, Summer | | Nagasaki, Fall | | Nagasaki, Winter | |
| --- | --- | --- | --- | --- | --- | --- | --- | --- |
| Category | Species | Ratio | Species | Ratio | Species | Ratio | Species | Ratio |
| Top 7 Abundant Bacteria | Proteobacteria | 33.4% | Proteobacteria | 7.4% | Proteobacteria | 14.5% | Proteobacteria | 85.1% |
|  | Firmicutes | 14.2% | Firmicutes | 2.3% | Firmicutes | 11.5% | Actinobacteria | 7.2% |
|  | Actinobacteria | 6.9% | Actinobacteria | 0.9% | Actinobacteria | 7.2% | Firmicutes | 4.1% |
|  | Cyanobacteria | 1.0% | Bacteroidetes | 0.5% | Bacteroidetes | 1.7% | Cyanobacteria | 0.8% |
|  | Bacteroidetes | 0.8% | Cyanobacteria | 0.2% | Cyanobacteria | 0.5% | Bacteroidetes | 0.3% |
|  | Acidobacteria | 0.5% | Armatimonadetes | 0.1% | Acidobacteria | 0.1% | Verrucomicrobia | 0.1% |
|  | Gemmatimonadetes | 0.4% | Chloroflexi | 0.1% | Armatimonadetes | 0.1% | Armatimonadetes | 0.0% |
| Other Bacteria | - | 0.6% | - | 0.1% | - | 0.6% | - | 0.2% |
| Unclassified Bacteria | - | 42.3% | - | 88.6% | - | 63.8% | - | 2.1% |


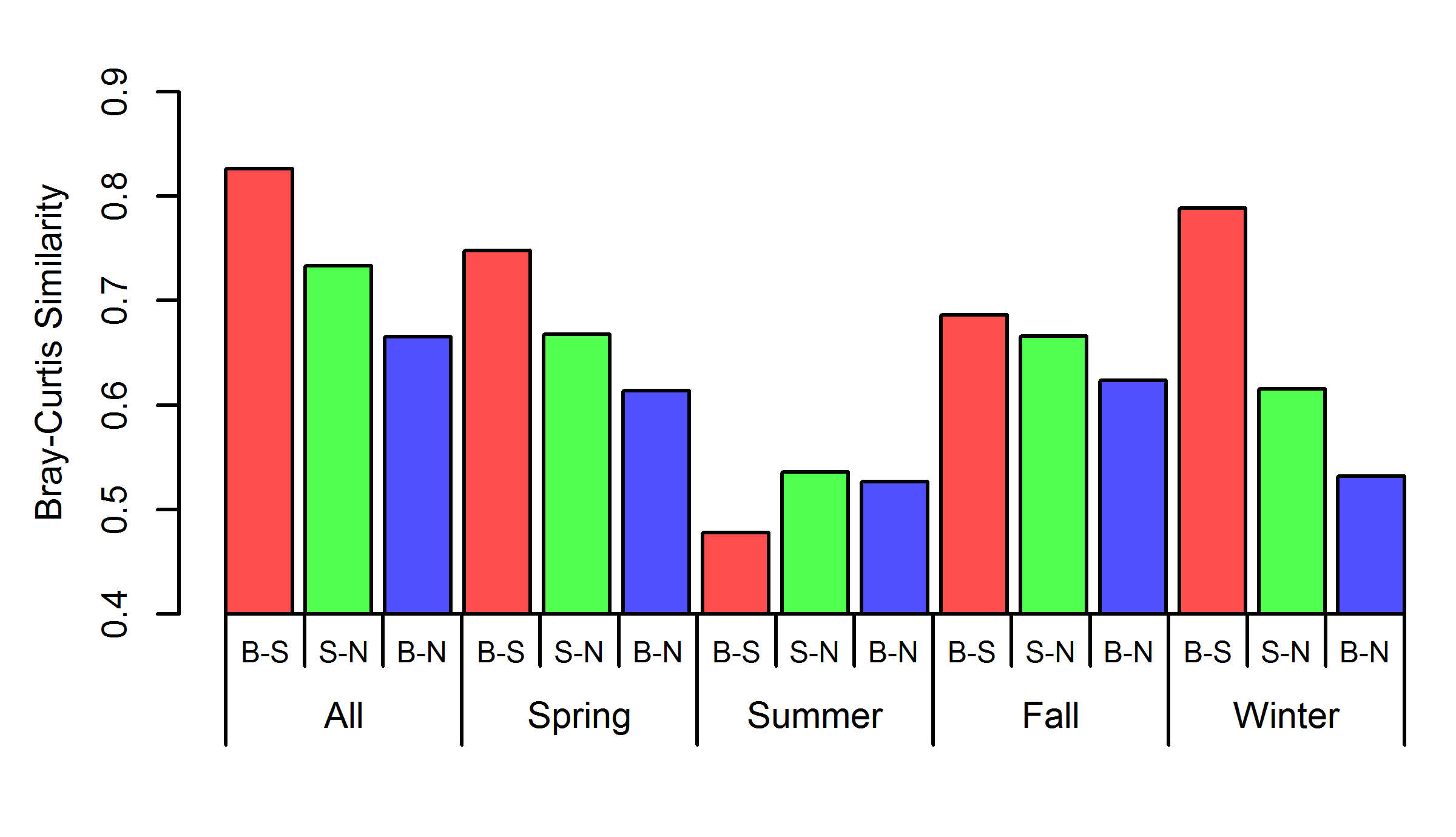


Figure S2. Bray-Curtis similarities of the identified phylotypes at the genus level between measurements sites. B-S, S-N, and B-N represent comparisons between Beijing and Seoul, Seoul and Nagasaki, and Beijing and Nagasaki, respectively.

Table S5. Description of collected samples and corresponding meteorological and environmental conditions.

| Location | Date | Diversity  (Genus) | PM_2.5_  (μg/m^3^) | Temperature  (°C) | Humidity (%) | Wind Speed (m/s) | Wind Direction (°) |
| --- | --- | --- | --- | --- | --- | --- | --- |
| Beijing | 2014-12-02 | 275 | 36.5 | -4.9 | 31.9 | 2.8 | - |
| Beijing | 2014-12-04 | 180 | 16.6 | -2.0 | 20.3 | 11.0 | 0 |
| Beijing | 2014-12-06 | 492 | 66.7 | -2.1 | 38.3 | 2.8 | - |
| Beijing | 2014-12-07 | 292 | 17.3 | 1.0 | 42.5 | 5.7 | 0 |
| Beijing | 2014-12-08 | 544 | 175.6 | -2.8 | 46.5 | 2.8 | - |
| Beijing | 2014-12-10 | 346 | 23.8 | -0.5 | 55.0 | 11.0 | 0 |
| Beijing | 2014-12-13 | 478 | 107.6 | -2.8 | 39.0 | 2.8 | - |
| Beijing | 2014-12-14 | 348 | 134.1 | -1.6 | 39.3 | 8.2 | 0 |
| Beijing | 2014-12-21 | 455 | 56.6 | -1.5 | 26.5 | 5.7 | 270 |
| Beijing | 2014-12-26 | 405 | 285.5 | -1.4 | 47.1 | 2.8 | - |
| Beijing | 2014-12-29 | 428 | 78.7 | 1.4 | 46.1 | 2.8 | - |
| Beijing | 2014-12-31 | 100 | 16.2 | -2.4 | 15.9 | 11.0 | 0 |
| Beijing | 2015-03-06 | 174 | 618.4 | 2.1 | 43.7 | 5.7 | 180 |
| Beijing | 2015-03-07 | 107 | 218.2 | 4.7 | 55.8 | 2.8 | - |
| Beijing | 2015-03-16 | 108 | 303.0 | 8.5 | 52.0 | 2.8 | - |
| Beijing | 2015-03-22 | 402 | 44.0 | 10.8 | 11.8 | 5.7 | 0 |
| Beijing | 2015-03-31 | 89 | 64.6 | 12.7 | 76.6 | 5.7 | 45 |
| Beijing | 2015-04-24 | 124 | 61.9 | 18.9 | 39.1 | 2.8 | - |
| Beijing | 2015-05-06 | 274 | 26.8 | 17.5 | 19.0 | 5.7 | 180 |
| Beijing | 2015-05-18 | 182 | 12.8 | 23.7 | 42.3 | 8.2 | 0 |
| Beijing | 2015-05-21 | 123 | 19.1 | 19.0 | 51.5 | 5.7 | 180 |
| Beijing | 2015-05-24 | 107 | 76.5 | 21.0 | 69.0 | 5.7 | 180 |
| Beijing | 2015-06-05 | 89 | 34.8 | 22.9 | 68.6 | 2.8 | - |
| Beijing | 2015-06-08 | 103 | 31.0 | 22.7 | 44.7 | 2.8 | - |
| Beijing | 2015-06-26 | 63 | 861.4 | 22.5 | 88.5 | 2.8 | - |
| Beijing | 2015-07-02 | 146 | 22.1 | 25.5 | 47.5 | 2.8 | - |
| Beijing | 2015-08-01 | 75 | 96.7 | 25.0 | 94.5 | 2.8 | - |
| Beijing | 2015-08-07 | 67 | 54.9 | 26.0 | 89.0 | 2.8 | - |
| Beijing | 2015-09-16 | 67 | 74.8 | 19.0 | 79.5 | 2.8 | - |
| Beijing | 2015-09-24 | 141 | 17.3 | 20.5 | 84.0 | 2.8 | - |
| Beijing | 2015-09-27 | 100 | 21.4 | 19.5 | 66.8 | 2.8 | - |
| Beijing | 2015-09-30 | 115 | 13.2 | 16.3 | 83.8 | 8.2 | 0 |
| Beijing | 2015-10-03 | 138 | 48.7 | 18.3 | 56.9 | 2.8 | - |
| Beijing | 2015-10-06 | 66 | 305.4 | 18.3 | 82.6 | 2.8 | - |
| Beijing | 2015-10-12 | 350 | 27.2 | 16.6 | 41.1 | 2.8 | - |
| Beijing | 2015-10-16 | 50 | 180.2 | 15.8 | 81.5 | 2.8 | - |
| Beijing | 2015-10-27 | 225 | 13.1 | 9.0 | 50.7 | 5.7 | 0 |
| Beijing | 2015-10-30 | 222 | 729.4 | 6.7 | 46.3 | 2.8 | - |
| Beijing | 2015-11-11 | 69 | 97.2 | 5.8 | 94.0 | 2.8 | - |
| Seoul | 2014-12-04 | 393 | 20.1 | -4.6 | 43.0 | 2.7 | 290 |
| Seoul | 2014-12-07 | 229 | 34.7 | -3.4 | 44.0 | 1.6 | 50 |
| Seoul | 2014-12-10 | 114 | 56.2 | 1.6 | 59.0 | 1.9 | 200 |
| Seoul | 2014-12-13 | 158 | 24.3 | -5.2 | 46.0 | 3.2 | 290 |
| Seoul | 2014-12-16 | 81 | 33.1 | -4.9 | 59.0 | 4.9 | 290 |
| Seoul | 2014-12-19 | 79 | 43.6 | -3.8 | 55.0 | 3.4 | 70 |
| Seoul | 2014-12-22 | 91 | 31.7 | -4.7 | 65.0 | 2.2 | 50 |
| Seoul | 2014-12-25 | 102 | 24.1 | -2.6 | 52.0 | 2.5 | 270 |
| Seoul | 2014-12-28 | 94 | 77.1 | -0.8 | 64.0 | 2.2 | 50 |
| Seoul | 2014-12-29 | 93 | 150.8 | 2.9 | 73.0 | 2.3 | 50 |
| Seoul | 2014-12-31 | 537 | 19.5 | -1.9 | 47.0 | 3.8 | 290 |
| Seoul | 2015-03-01 | 159 | 56.9 | 2.4 | 57.0 | 3.7 | 270 |
| Seoul | 2015-03-16 | 91 | 107.4 | 9.8 | 44.0 | 2.0 | 270 |
| Seoul | 2015-03-18 | 79 | 51.4 | 10.7 | 62.0 | 4.0 | 70 |
| Seoul | 2015-03-20 | 339 | 114.1 | 13.4 | 33.0 | 2.5 | 270 |
| Seoul | 2015-03-21 | 68 | 155.3 | 11.7 | 31.0 | 2.7 | 250 |
| Seoul | 2015-03-30 | 52 | 79.2 | 13.2 | 57.0 | 2.6 | 270 |
| Seoul | 2015-03-31 | 43 | 80.8 | 12.9 | 81.0 | 2.2 | 70 |
| Seoul | 2015-04-11 | 50 | 44.5 | 13.5 | 39.0 | 2.5 | 270 |
| Seoul | 2015-04-16 | 95 | 37.5 | 9.9 | 70.0 | 3.6 | 250 |
| Seoul | 2015-04-29 | 73 | 23.2 | 17.0 | 77.0 | 2.9 | 70 |
| Seoul | 2015-04-30 | 32 | 31.2 | 21.2 | 57.0 | 2.2 | 70 |
| Seoul | 2015-05-21 | 71 | 50.1 | 18.7 | 50.0 | 3.7 | 230 |
| Seoul | 2015-07-15 | 99 | 30.4 | 27.9 | 59.0 | 2.9 | 70 |
| Seoul | 2015-08-01 | 30 | 36.8 | 28.2 | 73.0 | 3.1 | 230 |
| Seoul | 2015-08-20 | 39 | 35.0 | 25.4 | 76.0 | 1.7 | 50 |
| Seoul | 2015-09-09 | 196 | 7.2 | 22.4 | 43.0 | 2.9 | 50 |
| Seoul | 2015-09-12 | 83 | 136.9 | 19.2 | 60.0 | 1.9 | 270 |
| Seoul | 2015-09-24 | 41 | 27.2 | 23.4 | 56.0 | 1.9 | 20 |
| Seoul | 2015-10-12 | 180 | 6.6 | 12.3 | 57.0 | 2.7 | 290 |
| Seoul | 2015-10-24 | 131 | 19.9 | 16.9 | 64.0 | 2.5 | 320 |
| Seoul | 2015-11-17 | 132 | 14.1 | 13.9 | 70.0 | 1.8 | 290 |
| Seoul | 2015-11-23 | 171 | 4.2 | 8.5 | 81.0 | 3.3 | 70 |
| Nagasaki | 2014-12-01 | 171 | 6.5 | 12.5 | 63.0 | 7.2 | 293 |
| Nagasaki | 2014-12-03 | 69 | 12.4 | 9.6 | 73.0 | 2.6 | 248 |
| Nagasaki | 2014-12-04 | 107 | 4.7 | 8.3 | 77.0 | 3.2 | 315 |
| Nagasaki | 2014-12-07 | 72 | 13.2 | 6.0 | 71.0 | 1.1 | 113 |
| Nagasaki | 2014-12-10 | 49 | 10.1 | 10.4 | 77.0 | 1.6 | 68 |
| Nagasaki | 2014-12-13 | 120 | 9.7 | 6.7 | 66.0 | 3.3 | 315 |
| Nagasaki | 2014-12-16 | 60 | 7.1 | 7.2 | 74.0 | 4.1 | 315 |
| Nagasaki | 2014-12-19 | 58 | 7.3 | 5.0 | 71.0 | 1.4 | 23 |
| Nagasaki | 2014-12-22 | 82 | 11.7 | 5.2 | 71.0 | 2.8 | 315 |
| Nagasaki | 2014-12-25 | 139 | 14.2 | 7.6 | 55.0 | 2.7 | 338 |
| Nagasaki | 2014-12-28 | 49 | 12.3 | 8.6 | 65.0 | 1.2 | 90 |
| Nagasaki | 2014-12-31 | 53 | 14.6 | 8.8 | 60.0 | 4.6 | 293 |
| Nagasaki | 2015-04-09 | 47 | 13.1 | 13.3 | 67.0 | 2.7 | 23 |
| Nagasaki | 2015-04-10 | 104 | 3.9 | 13.6 | 84.0 | 3.2 | 23 |
| Nagasaki | 2015-04-13 | 43 | 12.8 | 14.6 | 89.0 | 2.6 | 0 |
| Nagasaki | 2015-04-15 | 43 | 18.5 | 14.2 | 66.0 | 5.2 | 248 |
| Nagasaki | 2015-04-16 | 191 | 29.2 | 18.3 | 73.0 | 4.4 | 225 |
| Nagasaki | 2015-04-24 | 48 | 27.7 | 15.1 | 66.0 | 1.9 | 225 |
| Nagasaki | 2015-04-27 | 58 | 27.4 | 18.3 | 56.0 | 1.5 | 248 |
| Nagasaki | 2015-04-30 | 43 | 9.3 | 19.9 | 77.0 | 1.8 | 23 |
| Nagasaki | 2015-06-08 | 58 | 4.6 | 20.9 | 91.0 | 2.4 | 225 |
| Nagasaki | 2015-06-12 | 33 | 34.7 | 22.7 | 93.0 | 1.9 | 248 |
| Nagasaki | 2015-06-13 | 43 | 16.7 | 20.5 | 88.0 | 1.5 | 68 |
| Nagasaki | 2015-07-02 | 44 | 13.4 | 21.9 | 86.0 | 1.5 | 225 |
| Nagasaki | 2015-08-07 | 68 | 23.8 | 30.3 | 67.0 | 1.4 | 225 |
| Nagasaki | 2015-08-16 | 61 | 12.1 | 27.4 | 80.0 | 1.6 | 0 |
| Nagasaki | 2015-08-19 | 53 | 12.8 | 26.6 | 87.0 | 1.3 | 113 |
| Nagasaki | 2015-10-12 | 126 | 9.4 | 19.3 | 55.0 | 3.9 | 293 |
| Nagasaki | 2015-10-27 | 118 | 23.3 | 20.3 | 71.0 | 3.4 | 293 |
| Nagasaki | 2015-10-29 | 133 | 17.8 | 16.1 | 59.0 | 1.5 | 90 |
| Nagasaki | 2015-10-30 | 95 | 13.6 | 16.3 | 65.0 | 1.3 | 0 |
| Nagasaki | 2015-11-02 | 96 | 15.2 | 14.8 | 76.0 | 1.4 | 315 |
| Nagasaki | 2015-11-05 | 126 | 11.7 | 18.7 | 68.0 | 1.6 | 68 |

Table S6. Correlation coefficients between the Shannon diversity index of airborne bacterial communities and environmental/meteorological factors.

|  | **Daily** | **MA2** | **MA3** | **MA8** |
| --- | --- | --- | --- | --- |
| **Humidity (%)** | **-0.49** | -0.49 | -0.47 | -0.45 |
| **Wind Speed (m/s)** | 0.34 | 0.42 | 0.48 | **0.48** |
| **Temperature (°C)** | -0.30 | -0.30 | -0.30 | **-0.37** |

Note: MA2, MA3, and MA8 represent the 2-, 3-, and 8-day moving averages, respectively.


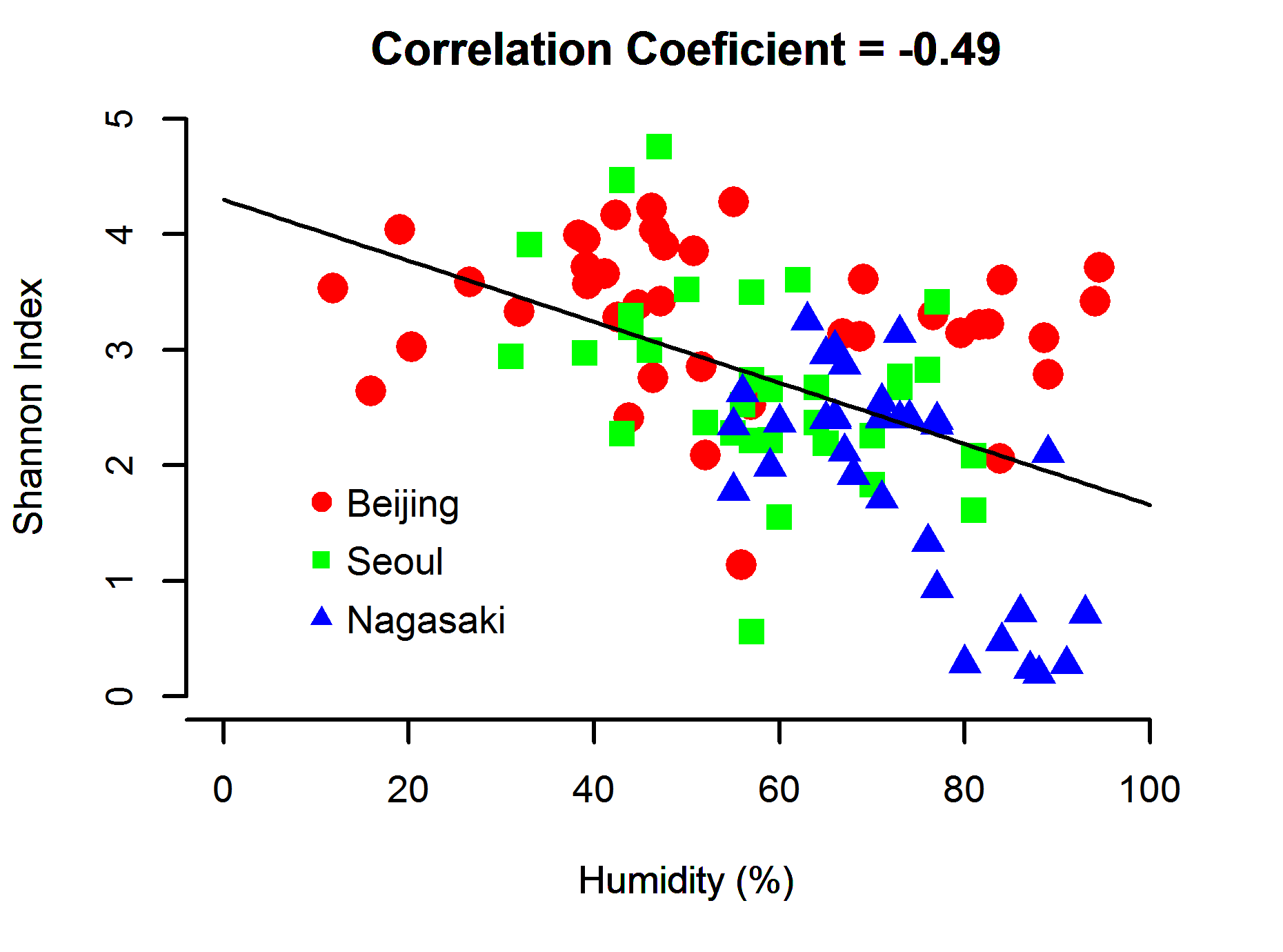

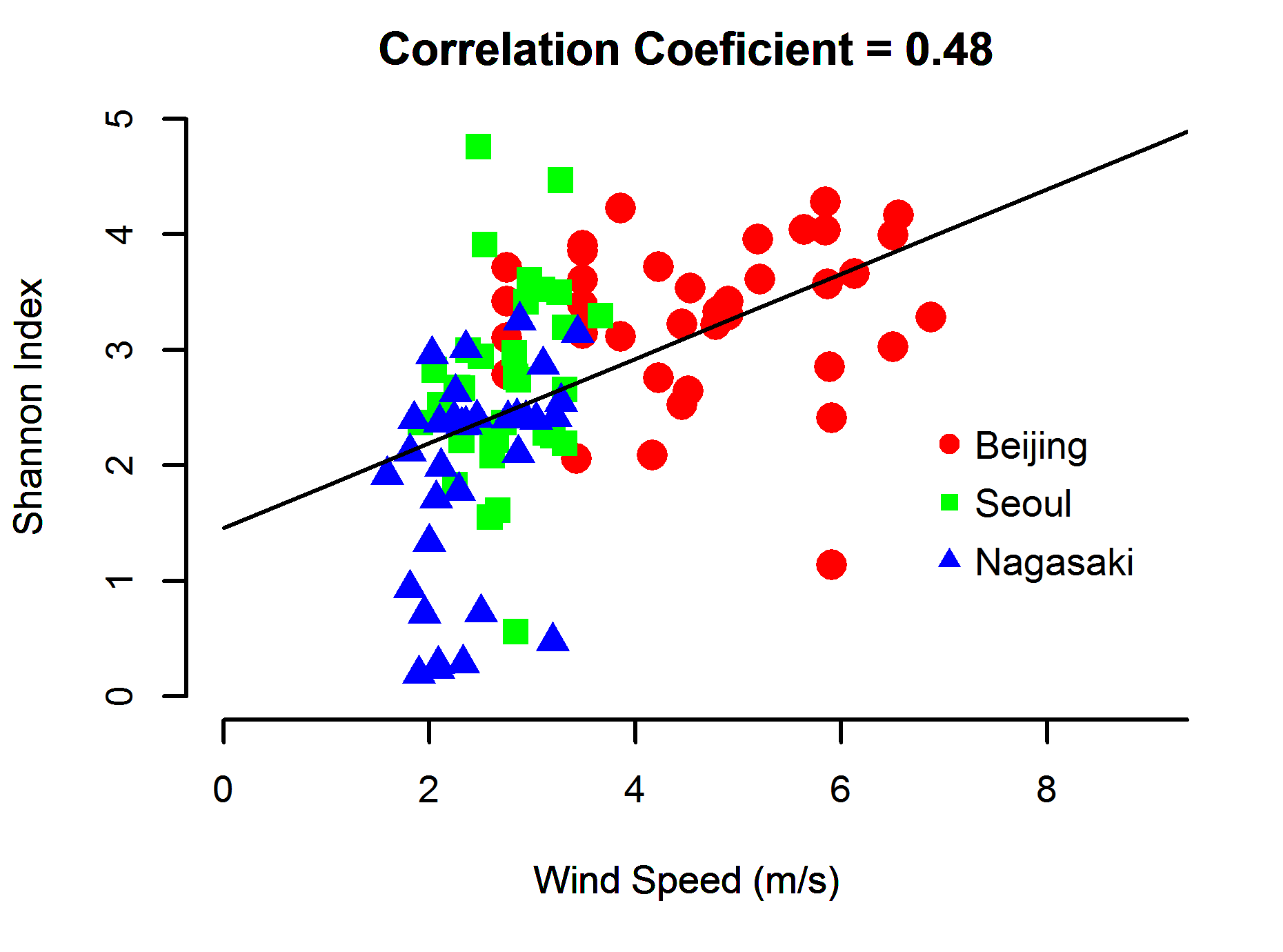


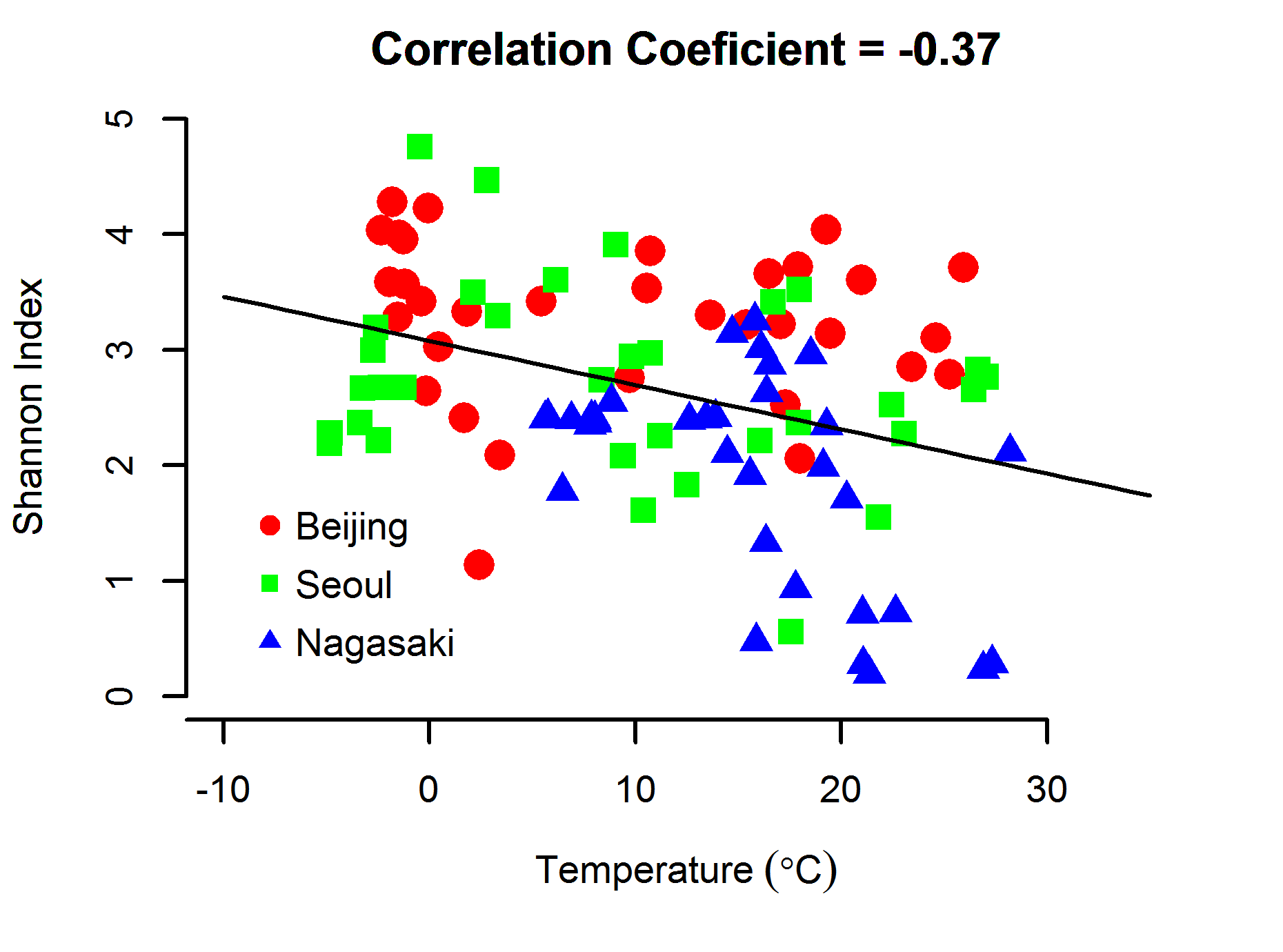


Figure S3. Scatter plots showing the correlation between airborne bacterial diversity and meteorological factors, such as humidity, wind speed, and temperature.
